# Local treatment of a bone graft by soaking in zoledronic acid inhibits bone resorption and bone formation. A bone chamber study in rats

**DOI:** 10.1186/1471-2474-13-240

**Published:** 2012-12-05

**Authors:** Ola Belfrage, Hanna Isaksson, Magnus Tägil

**Affiliations:** 1 Department of Orthopedics, Clinical Sciences, Lund University and Skåne University Hospital, Lund, S-221 85, Sweden; 2 Division of Solid Mechanics, Lund University, Lund, Sweden

**Keywords:** Zoledronic acid, Bisphosphonates, Allograft, Local treatment, Bone remodelling

## Abstract

**Background:**

Bone grafts are frequently used in orthopaedic surgery. Graft remodelling is advantageous but can occur too quickly, and premature bone resorption might lead to decreased mechanical integrity of the graft. Bisphosphonates delay osteoclastic bone resorption but may also impair formation of new bone. We hypothesize that these effects are dose dependent. In the present study we evaluate different ways of applying bisphosphonates locally to the graft in a bone chamber model, and compare that with systemic treatment.

**Methods:**

Cancellous bone grafts were placed in titanium chambers and implanted in the tibia of 50 male rats, randomly divided into five groups. The first group served as negative control and the grafts were rinsed in saline before implantation. In the second and third groups, the grafts were soaked in a zoledronic acid solution (0.5 mg/ml) for 5 seconds and 10 minutes respectively before being rinsed in saline. In the fourth group, 8 μL of zoledronic acid solution (0.5 mg/ml) was pipetted onto the freeze-dried grafts without rinsing. The fifth group served as positive control and the rats were given zoledronic acid (0.1 mg/kg) systemically as a single injection two weeks after surgery. The grafts were harvested at 6 weeks and analysed with histomorphometry, evaluating the ingrowth distance of new bone into the graft as an equivalent to the anabolic osteoblast effect and the amount (bone volume/total volume; BV/TV) of remaining bone in the remodelled graft as equivalent to the catabolic osteoclast effect.

**Results:**

In all chambers, almost the entire graft had been revascularized but only partly remodelled at harvest. The ingrowth distance of new bone into the graft was lower in grafts soaked in zoledronic acid for 10 minutes compared to control (p = 0.007). In all groups receiving zoledronic acid, the BV/TV was higher compared to control.

**Conclusions:**

This study found a strong inhibitory effect on bone resorption by bisphosphonates but also a limited inhibition of the ingrowth of new bone. Local treatment at surgery resulted in stronger inhibition of both resorption and bone formation compared to systemic treatment.

## Background

Morsellised bone grafts can be used in orthopaedic surgery both as autografts for biological support, to stimulate healing in fractures and non-unions, and as allografts for structural support in for example joint prosthesis revisions with osteolysis [1]. Regardless of origin and purpose, the bone graft is gradually revascularized and more or less remodelled into new bone to be incorporated in a new load-bearing bony structure [2]. Both the formation of new bone and resorption of bone are needed in bone graft remodelling and bone healing. Ideally, there is a balance between bone formation and bone resorption but sometimes the balance is skewed. The anabolic drive may fail due to absence of circulation, chemical signals like Bone Morphogenetic Proteins (BMPs) or cells to react to these signals [3]. Sometimes, a catabolic hyperactivity, due to instability or stress-shielding, might lead to graft resorption before new bone has replaced the allograft around a hip prosthesis [4]. Systemic bisphosphonates have been used successfully to balance an increased bone resorption both experimentally [5,6] as well as clinically [7].

Local administration of a bisphosphonate, directly to the graft is feasible and results in a rather strong chemical binding between the phosphate groups of the bisphosphonate and the hydroxyapatite crystals of bone [8]. In experimental studies, local intraoperative bisphosphonate have decreased the bone resorption after treatment at a fracture site [9] or in bone grafting [10]. However, in other studies using local treatment, a decrease was found also regarding the formation of new bone [11,12] and the authors speculated that a toxic effect of a too high dose of bisphosphonate would block bone metabolism, osseointegration and implant fixation.

The most commonly used way of local treatment of allograft with a bisphosphonate is to soak it in a solution containing the drug. The soaking time in different studies has varied between 3 and 10 minutes (Table 1). After soaking, the allograft is normally rinsed in saline to remove any unbound bisphosphonate before implantation since bisphosphonates bind chemically to bone.

**Table 1:** Local graft treatment with bisphosphonate

**Study**	**Drug**	**Concentration**	**Soaking time**	**Rinse**
Agholme 2009 [13]	Alendronate	2mg/ml	10 min	3x3 min/no
Aspenberg 2002 [10]	Alendronate	1 mg/ml	10 min	3x3 min
Baas 2008 [11]	Pamidronate	9mg/ml	3 min	No
Belfrage 2011 [6]	Zoledronate	0.5 mg/ml	Topical	No
Jakobsen 2007 [14]	Alendronate	2 mg/ml	3 min	No
Jakobsen 2010 [12]	Zoledronate	0.005/0.05/0.5 mg/ml	3 min	3 min
Jeppsson 2003 [15]	Clodronate	60 mg/ml	10 min	3x3 min
Kesteris 2006 [7]	Clodronate	60 mg/ml 10 ml	3 min	Washed 500 ml NaCl
Seo 2010 [16]	Zoledronate	30 μM, 10 ml	5 min	No
Tägil 2004 [5]	Alendronate	1 mg/ml	10 min	3x3 min
Tägil 2006 [17]	Zoledronate	0.7 mg sc	*	-

*Systemic injection of zoledronic acid to graft donor rat 24 hours before being euthanized.

Relevant studies of local treatment of bone graft with bisphosphonates listed by first author, bisphosphonate used, concentration of the drug, soaking time and rinsing procedure.

The aim of the present study was to evaluate how different doses and modes of administration, adopted from the clinical practise, influence both bone resorption and ingrowth of new bone into an allograft. We hypothesized that both local and systemic bisphosphonate treatment would lead to an increase in bone density but also that the bone ingrowth distance might be decreased by local but not systemic treatment. We also hypothesized that a longer soaking time would lead to denser bone and that topical administration without rinsing would have the strongest effect on both bone density and bone ingrowth.

## Methods

Cancellous bone allografts (n = 50) were placed in titanium bone chambers to later be implanted in the right proximal tibia of 50 rats. The grafts were randomly divided into five groups. The grafts in the control group were rinsed in saline, three local treatment groups received zoledronic acid (Zometa®, Novartis, North Ryde NSW, Australia) in three different local regimes and the grafts in the systemic group received systemic treatment with zoledronic acid (Table 2). The grafts were harvested at 6 weeks and analysed with histomorphometry.

**Table 2:** Treatment groups in the present study

**Group**	**Treatment**	**Rinse in saline**	**N**
**1**	Saline control	3 x 3 min	10
**2**	Zoledronic acid 0.5 mg/mL, short soaking time, 5 seconds	3 x 3 min	10
**3**	Zoledronic acid 0.5 mg/mL, long soaking time, 10 minutes	3 x 3 min	10
**4**	Topical zoledronic acid 4 μg, no rinse	None	10
**5**	Zoledronic acid systemic injection, 0.1 mg/kg	3 x 3 min	10

### The chamber

**Figure 1:**
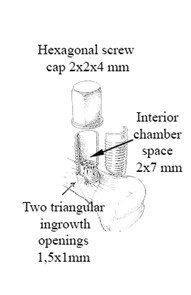
**Bone conduction chamber.** The chamber consists of a threaded titanium cylinder, formed out of 2 half-cylinders held together by a hexagonal screw cap. The interior of the chamber is 7 mm long with a diameter of 2 mm. One end of the implant is screwed into the proximal tibia. At this end, there are 2 ingrowth openings each measuring 0.75 mm^2^, where tissue can grow in from the subcortical bone.

The chamber consists of a threaded titanium cylinder, formed out of 2 half-cylinders held together by a hexagonal screw cap. The interior of the chamber is 7 mm long with a diameter of 2 mm. One end of the implant is screwed into the proximal tibia. At this end, there are 2 ingrowth openings each measuring 0.75 mm^2^, where tissue can grow in from the subcortical bone (Figure 1). Inside the bone chamber the cancellous allograft remodels in vivo. The graft is revascularized and remodelled from the base of the chamber and upward.

### Grafts and drug administration

Structurally intact cancellous bone grafts were obtained from female Sprague–Dawley rats (200 g; Taconic M&B A/S, Ry, Denmark). A cylindrical bone rod with a diameter of 2 mm was harvested in the axial direction from the knee joint with a hole cutter and the epiphysis was discarded. The rods were approximately 4–5 mm long. The grafts were kept sterile, freeze-dried for 24 h and frozen at -80°C.

Zoledronic acid was delivered as a powder and was diluted in saline to a concentration of 0.5 mg/ml, to correspond to previous experiment [6]. At surgery, the thawed grafts were placed in 3 ml of zoledronic acid solution for 5 seconds (group 2) or 10 minutes (group 3), and then rinsed 3 times for 3 minutes in 3 ml 0.9% NaCl (saline) to remove unbound zoledronic acid. The grafts belonging to the negative and positive controls (group 1 and 5) were placed in a saline solution and rinsed as described above. The procedure was blinded to the surgeon. After rinsing, the grafts were placed in the chambers. The chambers were inserted in the right tibia of the recipient animals. In group 4 (topical), the thawed freeze-dried grafts were placed in the chamber and 8 μL (4 μg zoledronic acid) of experimental solution was pipetted to the graft just before closing the chamber with the screw cap. These grafts were not rinsed. All grafts were placed with the denser, proximal end, towards the ingrowth openings of the chamber. Surgery was performed in random order. Systemic treatment in group 5 was administered as a single subcutaneous injection (0.1 mg/kg) with zoledronic acid at two weeks after surgery.

### Surgical procedure and animals

50 male Sprague–Dawley rats (315–405 g; Taconic M&B A/S, Ry, Denmark) were anesthetized with diazepam and pentobarbitalnatrium. Antibiotic prophylaxis was given as 12.5 mg dihydrostreptomycin and 10 mg procaine benzylpenicillin. Under aseptic conditions, a longitudinal incision was made over the anteromedial aspect of the right proximal tibial metaphysis. A hole was made with a drill and the chamber was screwed into position with the ingrowth holes situated subcortically. The wound was closed, leaving the entire chamber subcutaneous. Postoperatively, analgesic was given as 4.5 μg buprenorphine subcutaneously. All animal handling was approved by the regional animal research ethics committee (M70-06). Institutional guidelines for the care and treatment of experimental animals were followed. There were two rats in each cage and they had free access to water and feed.

### Evaluation

**Figure 2:**
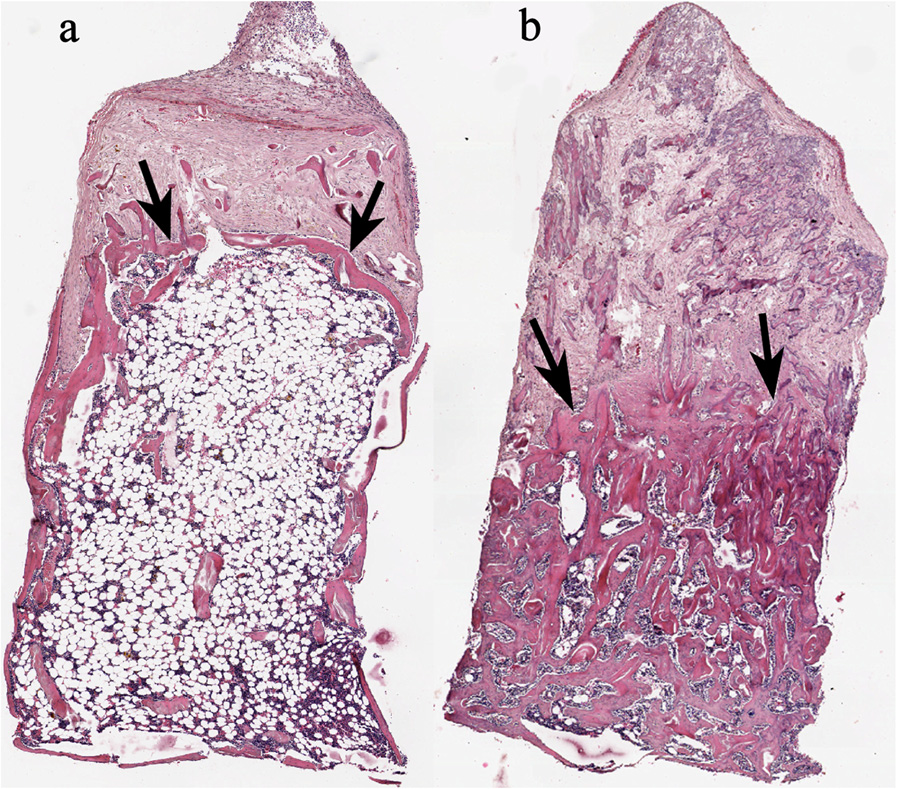
**Remodelled bone graft.** Inside the chamber, almost the entire graft has been revascularized at harvest with a fibrous tissue penetrating deepest into the graft and with the zone of new-forming bone (arrows) and resorbing osteoclasts lagging behind. Behind this layer of new-forming bone and close to the ingrowth openings, the new-formed bone as well as remnants of the graft is resorbed and replaced by a fatty bone marrow in the saline treated samples **(a)**. In the zoledronic acid treated grafts this marrow still consists of non-resorbed bone and both graft bone and newly formed bone are retained **(b)**. Hematoxyllin/Eosin x 4.

The rats were euthanized after 6 weeks and the contents of the chambers were prepared for histology. The specimens were fixed in 4% formaldehyde, and were decalcified, dehydrated, and embedded in paraffin. They were cut parallel to the long axis of the chamber with a microtome and stained with hematoxylin and eosin. Three sections from the middle of each specimen, each separated by 300 μm, were used for histological and histomorphometrical analyses. All sections were evaluated blindly and in random order. Host tissue enters the graft through holes in the bottom of the chamber and ingrowth/remodelling occurs from the bottom upwards. The front of bone ingrowth, the border between new-formed remodelled and non-remodelled bone is clearly visible (Figure 2). This ingrowth frontier together with the bottom and sides of the chamber forms the area of the newly formed remodelled bone. This new-formed remodelled bone area includes the new-formed marrow cavity within the graft as well as graft remnants that has been surrounded by new bone. In each slide this area was measured using a digital system and a digitizer (Videoplan; Kontron Bildanalyse, Esching, Germany) at 20× screen magnification. The mean bone ingrowth distance in each section was calculated by dividing new-formed remodelled bone area by the width of the specimen. The mean bone ingrowth distance for each animal was used as a surrogate variable of bone anabolism, *i.e.* how far into the graft the remodelling had taken place. The amount of remaining newly formed or graft bone behind the bone ingrowth frontier *i.e.* within the remodelled bone, was used as a surrogate variable of bone catabolism. A volume fraction estimation, equivalent to and expressed as BV/TV (bone volume/total volume), was made of both the remaining dead graft and living new-formed bone within this area by a point count method ad modum Cavalieri, using a Merz grid ocular lens [18] with 6 × 6 crossing lines forming 36 points. Multiple sections of the new-formed remodelled bone of each graft were analysed at 40× magnification using the point count method, from the bottom of the chamber to the bone ingrowth frontier. The frequency of the crossings covering graft and newly formed bone tissue was recorded and expressed as a percentage of the total area measured.

### Statistics

Normal distribution could not be assumed. Statistical analyses were performed using non-parametrical Mann Whitney *U*-test and Kruskal-Wallis test. The results were presented as medians and range. The data were analysed using SPSS software version 17.0 for Windows. Statistical significance was set at *p* < 0.05.

## Results

### Histology

**Figure 3:**
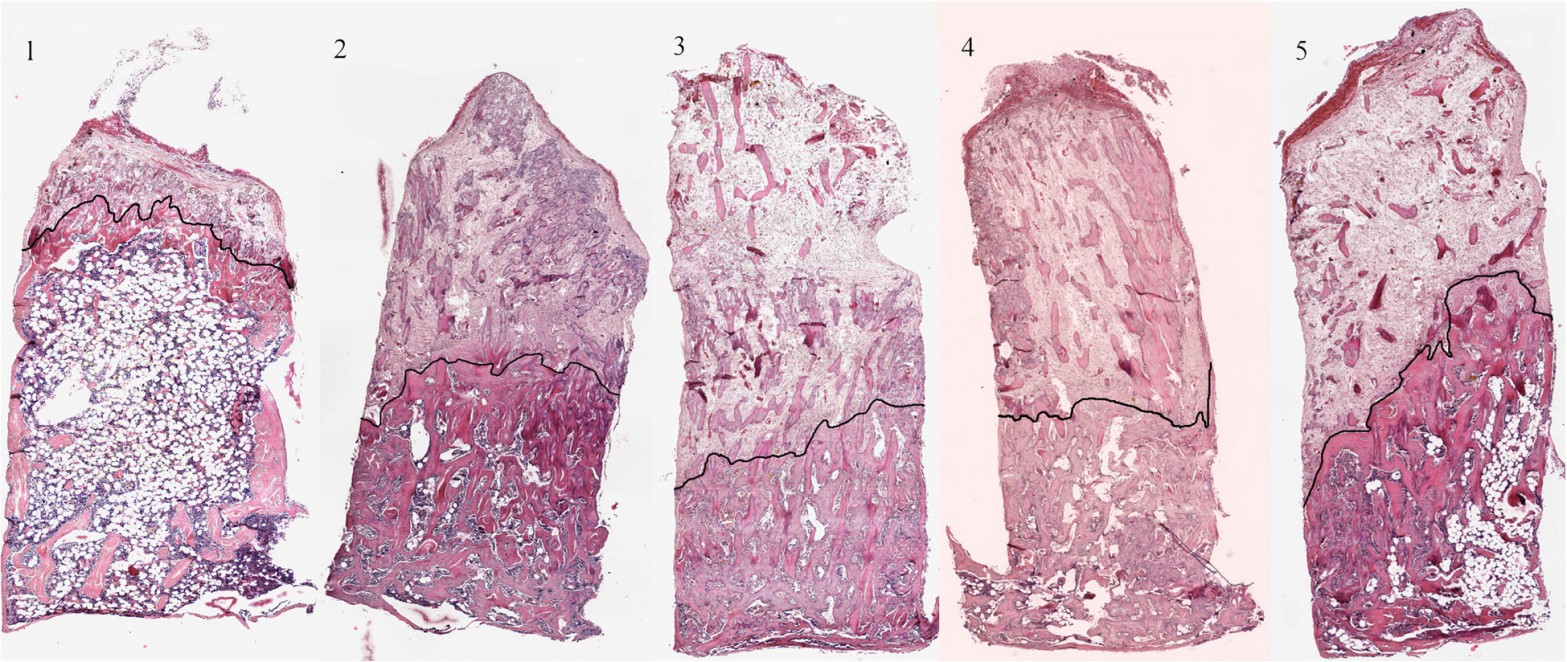
**Representative histological sections.** Representative histological sections of the five groups at 6 weeks. 1/Saline control. 2/Short soaking time. 3/Long soaking time. 4/Topical zoledronic acid, no rinse. 5/Zoledronic acid systemic injection at two weeks. Specimens are chosen to be representative of both median bone ingrowth distance and median bone density. The black line indicates the frontier between non-remodelled and remodelled graft bone and the bone ingrowth distance was less in group 3 compared to the other groups. Histologically, the increased bone volume fraction in the remodelled area can clearly be seen in all bisphosphonate treated groups.

No wound infection occurred. One rat in group 3 (long soaking time) was excluded due to misplacement of the chamber and complete lack of ingrowth and remodelling. In all chambers, almost the entire graft had been revascularized at harvest with fibrous tissue penetrating deep into the graft, with the zone of new-forming bone and resorbing osteoclasts lagging behind (Figure 2). Behind this layer of new-forming bone and close to the ingrowth openings, the new-formed bone as well as remnants of the graft was resorbed and replaced by a fatty bone marrow in the saline treated samples (Figure 2a). In the zoledronic acid treated grafts this marrow still consisted of bone (Figure 2b) and it appeared that both graft bone and newly formed bone was retained. Bone density seemed higher in the locally treated grafts with no fat present whereas some fatty bone marrow was seen in the systemically treated group. Bone ingrowth distance seemed shorter in the locally treated groups compared to control (Figure 3).

### Histomorphometry

**Figure 4:**
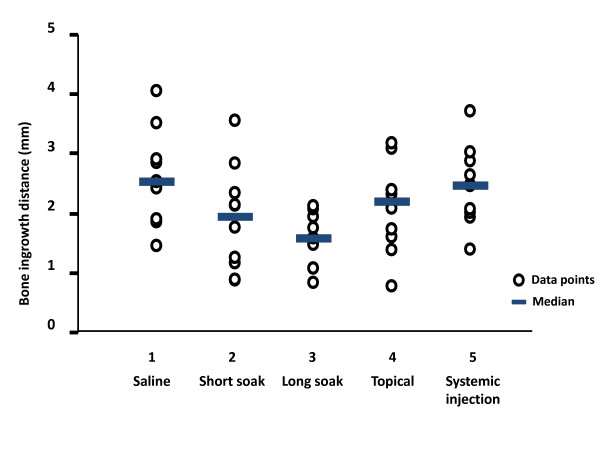
**Bone ingrowth distance.** Ingrowth distance (mm) of new bone into the allograft bone in the bone chamber after 6 week as assessed by histomorphometric analyses in each of the five experimental groups. Individual data points and the median for each group are indicated.

**Figure 5:**
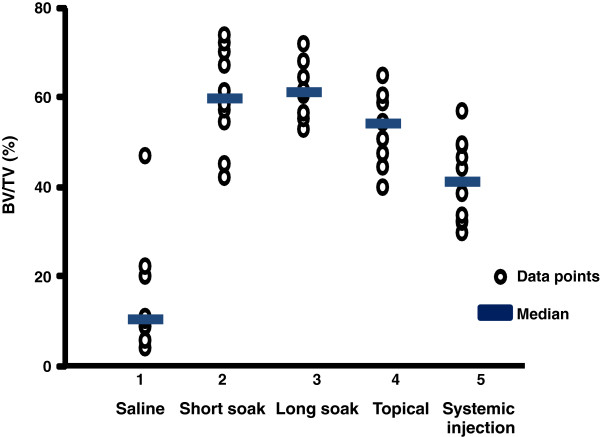
**Bone Volume/Total Volume.** Bone Volume/Total Volume (BV/TV, %) in the remodelled part of the allograft bone in the bone chamber after 6 week as assessed by histomorphometric analyses in each of the five experimental groups. Individual data points and the median for each group are indicated.

*Bone ingrowth distance;* the analysis of variance by ranks indicated a difference between groups (p = 0.02). The new-forming bone had penetrated the graft 2.5 mm (range 1.5-4.1 mm) in the saline group, and 2.5 mm (1.4-3.7 mm) in the systemically treated group. The group with a long soaking time in zoledronic acid showed a decreased ingrowth (median 1.6 mm, range 0.8-2.1 mm) compared to saline control (p = 0.007) and the systemic group (p = 0.008). In the other two local treatment groups, ingrowth was 2.0 (0.9-3.6) mm (short soaking time) and 2.2 (0.8-3.2) mm (topical) (Tables 3 and 4, Figure 4). *Bone density;* the analysis of variance by ranks indicated a strong difference between groups (p < 0.001). The relative amount of bone (BV/TV) in the remodelled part of the graft bone in the chamber was used to measure inhibition of bone resorption. BV/TV was 11% (range 4-47%) in the saline controls and 41% (30-57%) in the group receiving systemic treatment (p = 0.001 compared to saline control). BV/TV was 60% (42-74) and 61% (53-68) in the short soaking time and long soaking time groups. These differences were significant compared to both saline and systemic control. The topical group without rinsing had significantly lower BV/TV (54%) compared to the long soaking time group (p = 0.022) but higher compared to both control groups (p <0.001 and p = 0.01, respectively) (Tables 5 and 6, Figure 5). In all five groups the graft bone/new bone ratio was similar and 18-29% of the total amount of bone consists of graft bone (Tables 5 and 6).

**Table 3:** Bone ingrowth distance

**Group**	**Median ingrowth distance (mm)**	**Range (mm)**
1. Saline control, n = 10	2.5	1.5-4.1
2. Short soaking time, n = 10	2.0	0.9-3.6
3. Long soaking time, n = 9	1.6	0.8-2.1
4. Topical zoledronic acid, no rinse, n = 10	2.2	0.8-3.2
5. Zoledronic acid systemic injection, n = 10	2.5	1.4-3.7

Median bone ingrowth distance of new-formed bone into the chambers.

**Table 4:** Bone ingrowth distance

**Group**	**1. Saline control**	**2. Short soaking time**	**3. Long soaking time**	**4. Topical zoledronic acid, no rinse**	**5. Zoledronic acid systemic injection**
1. Saline control	-	-	-	-	-
2. Short soaking time	0.059	-	-	-	-
3. Long soaking time	**0.007**	0.25	-	-	-
4. Topical zoledronic acid, no rinse	0.13	0.55	0.086	-	-
5. Zoledronic acid systemic injection	0.82	0.11	**0.008**	0.29	-

P values for comparisons of bone ingrowth distance between treatment groups. Bold numbers indicate p < 0.05.

**Table 5:** Bone Volume/Total Volume (BV/TV)

**Group**	**Median BV/TV (%)**	**Range (%)**	**Ratio graft bone/total bone (%)**
1. Saline control, n = 10	11	4-47	23
2. Short soaking time, n = 10	60	42-74	29
3. Long soaking time, n = 9	61	53-68	18
4. Topical zoledronic acid, no rinse, n = 10	54	40-65	24
5. Zoledronic acid systemic injection, n = 10	41	30-57	25

Resorption of bone expressed as the amount of remaining bone in the remodelled area of the graft. The ratio between old graft bone and the total amount of bone including the newly formed bone is also presented.

**Table 6:** Bone Volume/Total Volume (BV/TV)

**Group**	**1. Saline control**	**2. Short soaking time**	**3. Long soaking time**	**4. Topical zoledronic acid, no rinse**	**5. Zoledronic acid systemic injection**
1. Saline control	-	-	-	-	-
2. Short soaking time	**<0.001**	-	-	-	-
3. Long soaking time	**<0.001**	1	-	-	-
4. Topical zoledronic acid, no rinse	**<0.001**	0.082	**0.022**	-	-
5. Zoledronic acid systemic injection	**0.001**	**0.002**	**0.001**	**0.01**	-

P values for comparisons of BV/TV between treatment groups. Bold numbers indicate p < 0.05.

## Discussion

### Bone formation

In designing the present experiment, one of the hypotheses was that unbound excessive bisphosphonate would interfere with bone formation and would therefore result in a decreased ingrowth distance, especially in the topical group that was not rinsed from excessive unbound bisphosphonate. A limited inhibition of the ingrowth of new bone was seen in all groups with the locally applied bisphosphonate, compared to both the saline and systemic bisphosphonate control groups. However, it was the rinsed group with the long soaking time that had a significantly decreased ingrowth distance and not the topical group as hypothesized. A decreased ingrowth caused by local treatment has not been shown in previous studies using the bisphosphonate alendronate in this model [10,13]. Alendronate is a potent bisphosphonate with a slightly lower affinity to bone and a lower antiresorptive effect compared to zoledronic acid [8]. In the above mentioned study by Agholme, where the alendronate concentration was higher, there was a weak trend for the bone ingrowth to be reduced in the overdose group, and although the difference was non-significant this could simply represent a power problem related to the fact that the animals were harvested at four weeks compared to six weeks in our study. Little is known about the effect of bisphosphonates on bone formation. In vitro, bisphosphonates decrease the viability of cultured osteoblasts in a dose dependent way [19-21]. Apoptosis is induced in a wide range of cells, and not only in osteoclasts [22,23]. Local tissue concentrations in vivo after systemic treatment are probably never as high as in the experimental conditions of the in vitro experiments [24], but might be when the graft is soaked in bisphosphonate solution. It has been speculated that the reduced bone formation using local treatment is caused by unbound bisphosphonate being released, reaching high tissue concentrations also in vivo. Therefore rinsing the allograft after soaking has been proposed to decrease this effect on bone formation [11]*.* In a canine study [12], morsellised allografts were soaked in zoledronic acid at three different concentrations (0.005, 0.05 or 0.5 mg/mL). The grafts were rinsed in saline, and packed around a titanium implant. The amount of new bone around the implants was dose-dependent; greatest in the low-dose group but with no new bone formation observed in the high dose group. In our study the amount of bisphosphonate bound to the bone mineral seemed to be more important than unbound. Similar findings have been reported in another bone chamber study, comparing saline treated grafts with grafts soaked in alendronate, with or without subsequent rinsing [13]. In that study, the amount of alendronate implanted was estimated to be nearly 150 times higher in the unrinsed overdose group compared to the rinsed regular dose but rinsing the graft did not affect bone ingrowth distance. Histology was performed after one week to identify signs of any toxic effect, such as inflammatory cells and necrosis but the groups were similar. In both studies thus rinsing the allograft after bisphosphonate treatment seems to be of minor importance regarding the effects on bone formation, probably, due to a fast dilution of unbound bisphosphonate into the surrounding tissue. Instead, the amount of bound bisphosphonate, determined by the affinity to bone mineral of the bisphosphonate used, the concentration of the bisphosphonate solution and the soaking time, seems to be more important. Allograft bone have osteoconductive properties but also osteoinductive. BMP-7 is present and is released with increasing strain [25] together with other BMPs that are present in the bone matrix. When allograft bone is resorbed during remodelling, the matrix will be broken down and release growth factors inducing bone formation. If the resorption is restricted by bisphosphonates the release of growth factors will also be reduced and this could explain the decreased bone ingrowth distance.

### Resorption

Regarding resorption, we found a strong effect of the bisphosphonate compared to the saline controls, regardless of application mode. In this unloaded model, almost all of the graft as well as the newly formed bone had been resorbed in the saline controls and replaced by fat cell rich marrow in the remodelled part of the graft (Figure 2). All groups with bisphosphonate treatment had a large amount of retained bone, more marked in the locally treated groups compared to the systemic single dose bisphosphonate, indicating a larger inhibitory effect by the local application over systemic. In the present, as well as in previous studies [13], rinsing allografts before implantation seems to be of minor importance also regarding the *anti-resorptive* effects of the bisphosphonates. The group with the densest bone in our study was local treatment with a long soaking time of 10 minutes, equivalent to the high concentration group in the Jakobsen study [12]. The marked clinical effect of a soaking time of only 5 seconds is noteworthy, which could probably be due to the fact that the allografts used were small, cancellous and freeze-dried which facilitated absorption. Further research is needed to evaluate the effects of bisphosphonates on bone grafts in humans and in a mechanically loaded situation. The *in vivo* effect on osteoblasts in bone remodelling is also a topic of further research.

## Conclusions

In this study we found a strong effect on bone density by bisphosphonates but also inhibition of the ingrowth of new bone in grafts soaked for long time. Local treatment at surgery resulted in both denser bone and a reduced bone ingrowth distance compared to systemic treatment.

## Competing interests

No competing interest was declared for this study.

## Authors' contributions

OB participated in the design of the study, executed surgery, performed the statistical analysis and wrote the manuscript. HI helped with statistics and editing the manuscript. MT conceived of the study, and participated in its design and coordination and helped to draft the manuscript. All authors read and approved the final manuscript.

## Pre-publication history

The pre-publication history for this paper can be accessed here:

http://www.biomedcentral.com/1471-2474/13/240/prepub

## References

[B1] Leopold SS Jacobs JJ Rosenberg AG Cancellous allograft in revision total hip arthroplasty. A clinical review Clin Orthop Relat Res 2000 86 97 10.1097/00003086-200002000-00010 10693553

[B2] Linder L Cancellous impaction grafting in the human femur: histological and radiographic observations in 6 autopsy femurs and 8 biopsies Acta Orthop Scand 2000 71 543 552 10.1080/000164700317362154 11145379

[B3] Marsell R Einhorn TA The biology of fracture healing Injury 2011 42 551 555 10.1016/j.injury.2011.03.031 21489527 PMC3105171

[B4] Toms AD Barker RL Jones RS Kuiper JH Impaction bone-grafting in revision joint replacement surgery J Bone Joint Surg Am 2004 86-A 2050 2060 15342772 10.2106/00004623-200409000-00028

[B5] Tagil M Astrand J Westman L Aspenberg P Alendronate prevents collapse in mechanically loaded osteochondral grafts: a bone chamber study in rats Acta Orthop Scand 2004 75 756 761 10.1080/00016470410004157 15762268

[B6] Belfrage O Flivik G Sundberg M Kesteris U Tagil M Local treatment of cancellous bone grafts with BMP-7 and zoledronate increases both the bone formation rate and bone density: a bone chamber study in rats Acta Orthop 2011 82 228 233 10.3109/17453674.2011.566138 21434769 PMC3235296

[B7] Kesteris U Aspenberg P Rinsing morcellised bone grafts with bisphosphonate solution prevents their resorption. A prospective randomised double-blinded study J Bone Joint Surg Brit Vol 2006 88 993 996 10.1302/0301-620X.88B8.17457 16877594

[B8] Russell RG Watts NB Ebetino FH Rogers MJ Mechanisms of action of bisphosphonates: similarities and differences and their potential influence on clinical efficacy Osteoporos Int 2008 19 733 759 10.1007/s00198-007-0540-8 18214569

[B9] Greiner SH Wildemann B Back DA Alidoust M Schwabe P Haas NP Schmidmaier G Local application of zoledronic acid incorporated in a poly(D, L-lactide)-coated implant accelerates fracture healing in rats Acta Orthop 2008 79 717 725 10.1080/17453670810016768 18839381

[B10] Aspenberg P Astrand J Bone allografts pretreated with a bisphosphonate are not resorbed Acta Orthop Scand 2002 73 20 23 11928906 10.1080/000164702317281350

[B11] Baas J Elmengaard B Jensen TB Jakobsen T Andersen NT Soballe K The effect of pretreating morselized allograft bone with rhBMP-2 and/or pamidronate on the fixation of porous Ti and HA-coated implants Biomaterials 2008 29 2915 2922 10.1016/j.biomaterials.2008.03.010 18407348

[B12] Jakobsen T Baas J Bechtold JE Elmengaard B Soballe K The effect of soaking allograft in bisphosphonate: a pilot dose–response study Clin Orthop Relat Res 2010 468 867 874 10.1007/s11999-009-1099-9 19763718 PMC2816745

[B13] Agholme F Aspenberg P Experimental results of combining bisphosphonates with allograft in a rat model J Bone Joint Surg Br 2009 91 670 675 10.1302/0301-620X.91B5.21867 19407306

[B14] Jakobsen SS Danscher G Stoltenberg M Larsen A Bruun JM Mygind T Kemp K Soballe K Cobalt-chromium-molybdenum alloy causes metal accumulation and metallothionein up-regulation in rat liver and kidney Basic Clin Pharmacol Toxicol 2007 101 441 446 10.1111/j.1742-7843.2007.00137.x 17971067

[B15] Jeppsson C Astrand J Tagil M Aspenberg P A combination of bisphosphonate and BMP additives in impacted bone allografts Acta Orthop Scand 2003 74 483 489 10.1080/00016470310017839 14521303

[B16] Seo SW Cho SK Storer SK Lee FY Zoledronate reduces unwanted bone resorption in intercalary bone allografts Int Orthop 2010 34 599 603 10.1007/s00264-009-0748-7 19343345 PMC2903128

[B17] Tagil M Aspenberg P Astrand J Systemic zoledronate precoating of a bone graft reduces bone resorption during remodeling Acta Orthop 2006 77 23 26 10.1080/17453670610045650 16534698

[B18] Merz WA Schenk RK A quantitative histological study on bone formation in human cancellous bone Acta Anat (Basel) 1970 76 1 15 10.1159/000143476 4099686

[B19] Orriss IR Key ML Colston KW Arnett TR Inhibition of osteoblast function in vitro by aminobisphosphonates J Cell Biochem 2009 106 109 118 10.1002/jcb.21983 19003973

[B20] Idris AI Rojas J Greig IR Van AMP Apos T Hof RJ Ralston SH Aminobisphosphonates Cause Osteoblast Apoptosis and Inhibit Bone Nodule Formation In Vitro Calcif Tissue Int 2008 82 3 191 201 10.1007/s00223-008-9104-y 18259679

[B21] Greiner S Kadow-Romacker A Lubberstedt M Schmidmaier G Wildemann B The effect of zoledronic acid incorporated in a poly(D, L-lactide) implant coating on osteoblasts in vitro J Biomed Mater Res A 2007 80 769 775 17041912 10.1002/jbm.a.30950

[B22] Aft R Bisphosphonates in breast cancer: clinical activity and implications of preclinical data Clin Adv Hematol Oncol 2011 9 194 205 21475125

[B23] Bukowski JF Dascher CC Das H Alternative bisphosphonate targets and mechanisms of action Biochem Biophys Res Commun 2005 328 746 750 10.1016/j.bbrc.2004.11.075 15694409

[B24] Schindeler A Little DG Bisphosphonate action: revelations and deceptions from in vitro studies J Pharm Sci 2007 96 1872 1878 10.1002/jps.20904 17518363

[B25] Board TN Rooney P Kay PR Strain imparted during impaction grafting may contribute to bony incorporation: AN IN VITRO STUDY OF THE RELEASE OF BMP-7 FROM ALLOGRAFT J Bone Joint Surg Br 2008 90-B 821 824 10.1302/0301-620X.90B6.20234 18539680

